# Interferon-α Regulates Glutaminase 1 Promoter through STAT1 Phosphorylation: Relevance to HIV-1 Associated Neurocognitive Disorders

**DOI:** 10.1371/journal.pone.0032995

**Published:** 2012-03-30

**Authors:** Lixia Zhao, Yunlong Huang, Changhai Tian, Lynn Taylor, Norman Curthoys, Yi Wang, Hamilton Vernon, Jialin Zheng

**Affiliations:** 1 Laboratory of Neuroimmunology and Regenerative Therapy, University of Nebraska Medical Center, Omaha, Nebraska, United States of America; 2 Departments of Pharmacology and Experimental Neuroscience, University of Nebraska Medical Center, Omaha, Nebraska, United States of America; 3 Departments of Pathology and Microbiology, University of Nebraska Medical Center, Omaha, Nebraska, United States of America; 4 Department of Biochemistry and Molecular Biology, Colorado State University, Fort Collins, Colorado, United States of America; University Hospital Zurich, Switzerland

## Abstract

HIV-1 associated neurocognitive disorders (HAND) develop during progressive HIV-1 infection and affect up to 50% of infected individuals. Activated microglia and macrophages are critical cell populations that are involved in the pathogenesis of HAND, which is specifically related to the production and release of various soluble neurotoxic factors including glutamate. In the central nervous system (CNS), glutamate is typically derived from glutamine by mitochondrial enzyme glutaminase. Our previous study has shown that glutaminase is upregulated in HIV-1 infected monocyte-derived-macrophages (MDM) and microglia. However, how HIV-1 leads to glutaminase upregulation, or how glutaminase expression is regulated in general, remains unclear. In this study, using a dual-luciferase reporter assay system, we demonstrated that interferon (IFN) α specifically activated the glutaminase 1 (GLS1) promoter. Furthermore, IFN-α treatment increased signal transducer and activator of transcription 1 (STAT1) phosphorylation and glutaminase mRNA and protein levels. IFN-α stimulation of GLS1 promoter activity correlated to STAT1 phosphorylation and was reduced by fludarabine, a chemical that inhibits STAT1 phosphorylation. Interestingly, STAT1 was found to directly bind to the GLS1 promoter in MDM, an effect that was dependent on STAT1 phosphorylation and significantly enhanced by IFN-α treatment. More importantly, HIV-1 infection increased STAT1 phosphorylation and STAT1 binding to the GLS1 promoter, which was associated with increased glutamate levels. The clinical relevance of these findings was further corroborated with investigation of post-mortem brain tissues. The glutaminase C (GAC, one isoform of GLS1) mRNA levels in HIV associated-dementia (HAD) individuals correlate with STAT1 (p<0.01), IFN-α (p<0.05) and IFN-β (p<0.01). Together, these data indicate that both HIV-1 infection and IFN-α treatment increase glutaminase expression through STAT1 phosphorylation and by binding to the GLS1 promoter. Since glutaminase is a potential component of elevated glutamate production during the pathogenesis of HAND, our data will help to identify additional therapeutic targets for the treatment of HAND.

## Introduction

HAND develop during progressive HIV-1 infection and are characterized by cognitive impairments, behavioral disorders and potential progressive motor abnormalities as a consequence of neuronal damage during prolonged inflammation in the CNS [1]. Activated macrophages and microglia are critical to the pathogenesis of HAND, as they are known to contribute to neuronal injury through the production and release of various soluble neurotoxic factors including glutamate [2], [3], [4]. Although glutamate is a neurotransmitter involved in vital physiologic processes, excess extracellular glutamate can lead to neuronal damage and death [5]. Specifically relevant to HAND, HIV-1 infected macrophages are a great source of extracellular glutamate [6], [7], however, the mechanisms of excess glutamate levels associated with HIV-1 infection remain unclear.

Our previous work has demonstrated that HIV-1-infected MDM and microglia produce more extracellular glutamate than uninfected cells [8], [9], an effect that is dependent on mitochondrial glutaminase [10]. In the CNS, mitochondrial glutaminase is a key enzyme that converts glutamine to glutamate, an enzymatic process that has the potential to cause neuronal toxicity [8], [11]. Kidney-type glutaminase (KGA) is highly expressed in the brain [12]; it has various isoforms generated through tissue-specific alternative splicing, including GAC, a KGA variant [13]. Human GAC mRNA is produced by alternative splicing of GLS1 gene, joining exons 1–15, while the human KGA mRNA has exons 1–14 and 16–19; the resulting protein of human GAC shares much of the functional human KGA regions (exon 1–14), but contains an unique C-terminal [14]. Furthermore, the human GAC isoform is specifically upregulated in MDM and microglia during HIV infection [9], [15]. Despite extensive studies of upstream molecular stimuli, particularly those that are responsible for HIV-1-mediated GAC upregulation and glutamate overproduction, are still obscure.

HIV-1 invasion of the CNS and infection of macrophages and microglia may lead to cellular activation and subsequent release of cytokines, chemokines, and growth factors that include type I IFNs, IFN-α and IFN-β [16]. All type I IFNs interact with the IFN-α receptor (IFNAR) [17], which couples to a uniform signal transduction cascade. IFN-IFNAR binding triggers receptor dimerization and activation [18], leading to phosphorylation of a tyrosine residue on IFNAR [19], tyrosine kinase JAK1/2 [20] and STAT [21], [22], ultimately leading to the dimerization of STAT [23]. Activated STAT dimers then dissociate from the receptor, translocate to the nucleus and bind to the interferon stimulated response elements (ISRE) of IFN-stimulated genes [24]. Our previous study shows that type I IFNs regulate the STAT pathway in HIV-1-infected MDM [25]; this innate immune response has been increasingly recognized to associate with the progression of HAND [26].

Regulation of glutaminase promoter activity remains to be fully elucidated. Rat GLS1 promoter was previously cloned and functionally analyzed in 2001 [27], [28]. Furthermore, human GLS2 promoter was described in 2003 [29]. In the present report, we cloned and characterized the human GLS1 promoter. Using a MDM model, we studied the mechanism of transcriptional activation of GLS1 specifically by IFN-α. We found that IFN-α induced STAT1 phosphorylation and subsequently enhanced STAT1 binding to the GLS1 promoter. More importantly, HIV-1 infection induced STAT1 to bind to the GLS1 promoter thereby leading to glutamate overproduction. Understanding glutaminase regulation and its effect on glutamate production in HIV-1 infection may elucidate further information on how HIV-1 infection induces neurotoxicity, ultimately helping to develop new therapies.

## Results

### IFN-α Specifically Activates the GLS1 Promoter

**Figure 1:**
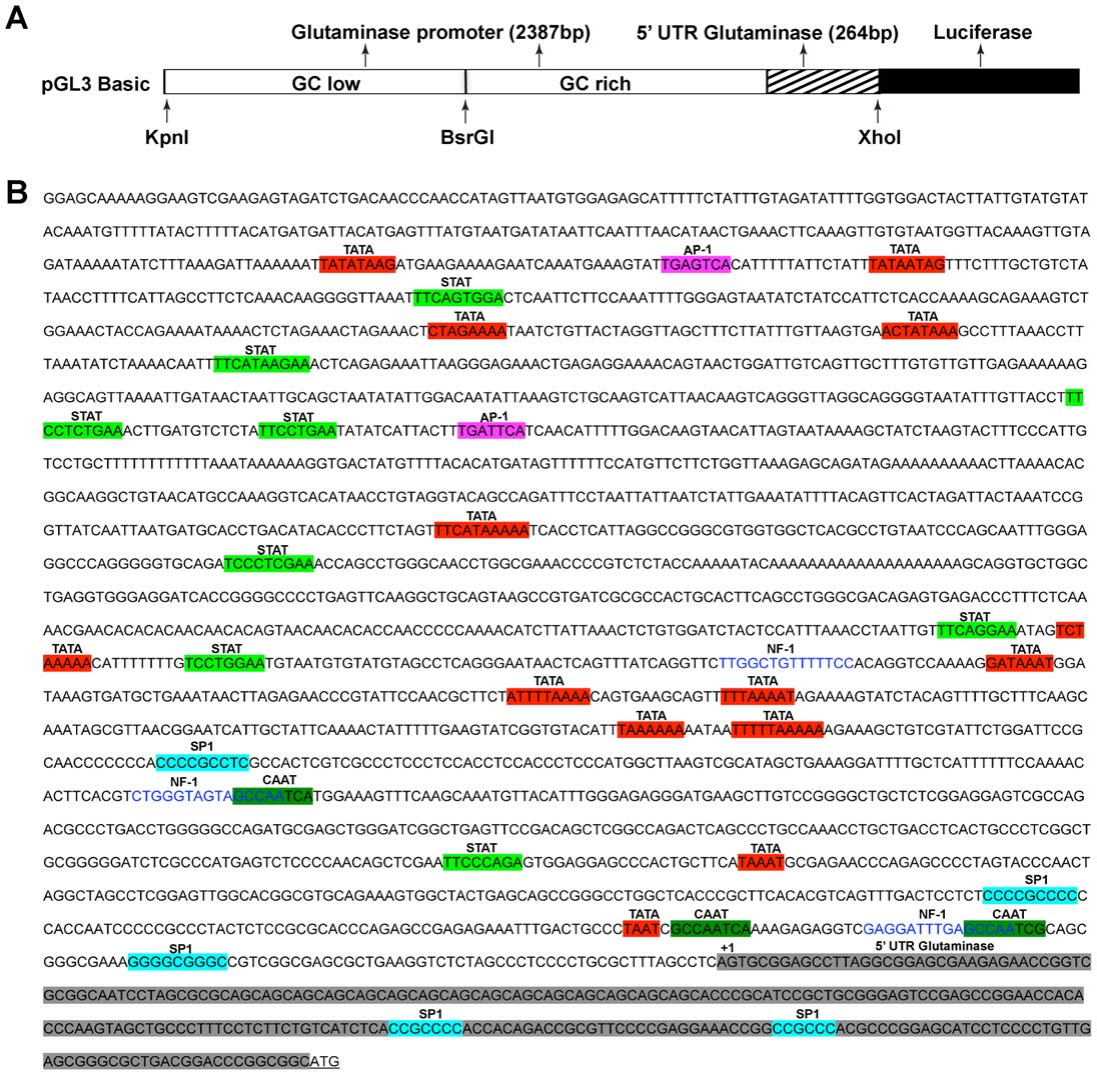
Human GLS1 promoter construct and sequence analysis. (A). Schematic diagram of the human GLS1 promoter-driven luciferase reporter construct. The construct contains the Firefly luciferase coding sequence (black) with regions of the promoter (blank) and the 5' UTR (shaded). The restriction enzymes used for cloning are shown. (B). Sequence analysis of the human GLS1 promoter. The TSS is designated as +1. The 5' UTR is shown in gray. The coding sequence is underlined. Putative transcription factor binding sites along the promoter sequence are highlighted in different colors, with their corresponding transcription factors shown on top of the sequence. Highlighted in red: TATA box, dark green: CAAT box, pink: AP-1, blue: SP-1, bright green: STAT, grey: 5' UTR glutaminase; font color in blue: NF-1.

To study the gene regulation of human glutaminase, we first looked at the GLS1 promoter. A GLS1 promoter construct was created from a BAC clone PR11-413B20 and ligated into a dual-luciferase system vector, pGL-3 basic (Fig. 1A). Analysis of the promoter sequence revealed putative binding sites for a number of transcription factors (Fig. 1B). Notably, the hallmark TATA box, which is usually located in the region of –25 to –30, was found at -111 to –108 (TAAT), with a putative CAAT box at –76 to –72. Other transcription factors that have the potential to bind to the promoter include activator protein 1 (AP-1), nuclear factor 1 (NF-1) and specificity protein 1 (SP-1), which are typically constitutively active in cells. When we looked for the inducible transcription factors, we found up to 8 sequences predicted to be STAT binding sites, indicating the factors that regulate STAT activation might have the potential to regulate the GLS1 promoter.

**Figure 2:**
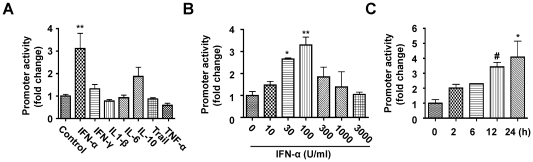
IFN-α specifically activates human GLS1 promoter. HEK 293T cells were co-transfected with the human GLS1 promoter construct and pRL-SV40. 24 hours later, the cells were treated with either various individual cytokines for another 24 hours (A), IFN-α of varying doses for 24 hours (B), or with 100 U/ml IFN-α for varied time lengths (C). In (A), 100 U/ml IFN-α, 100 ng/ml IFN-γ, 10 ng/ml IL-1β, 10 ng/ml IL-6, 25 ng/ml IL-10, 50 ng/ml TRAIL, or 50 ng/ml TNF-α was used. Luciferase activity in the lysates was measured by luminescence detection. Renilla luciferase was used as internal control to normalize transfection efficiency. The data are representative of three independent experiments and are the means of triplicate samples. #, p<0.05, *, p<0.01, **, p<0.001 in comparison to control.

Next, we used a panel of cytokines in the stimulation of cells to determine the possible factors that regulate the GLS1 promoter. We transiently co-transfected HEK 293T cells with GLS1 promoter-driven Firefly luciferase reporter plasmid and pRL-SV40 that served as an internal control for transfection efficiency. Of all the cytokines used, including IFN-α (100 U/ml), IFN-γ (100 ng/ml), IL-1β (10 ng/ml), IL-6 (10 ng/ml), IL-10 (25 ng/ml), TRAIL (50 ng/ml) and TNF-α (50 ng/ml), only IFN-α significantly increased (2-3-fold) the human GLS1 promoter activity (Fig. 2A). To confirm the effect of IFN-α, we examined different doses of IFN-α treatment and performed a time course to determine the effect on GLS1 promoter activity. We found that the increase in GLS1 promoter activity by IFN-α is dose-dependent (Fig. 2B) from 0 to 100 U/ml and time-dependent (Fig. 2C) from 0 to 24 hours. The effect of IFN-α on GLS1 promoter activity is specific because blocking IFN-α with neutralizing antibodies abolished IFN-α-induced GLS1 promoter activation (Fig. S2). Similarly, rat IFN-α activated the glutaminase promoter in rat astrocytes, suggesting regulation of glutaminase promoter by IFN-α is not limited to humans (Fig. S1A-C).

### IFN-α Induces STAT1 Phosphorylation and Subsequently Increases Glutaminase Expression and Glutamate Production in MDM

**Figure 3:**
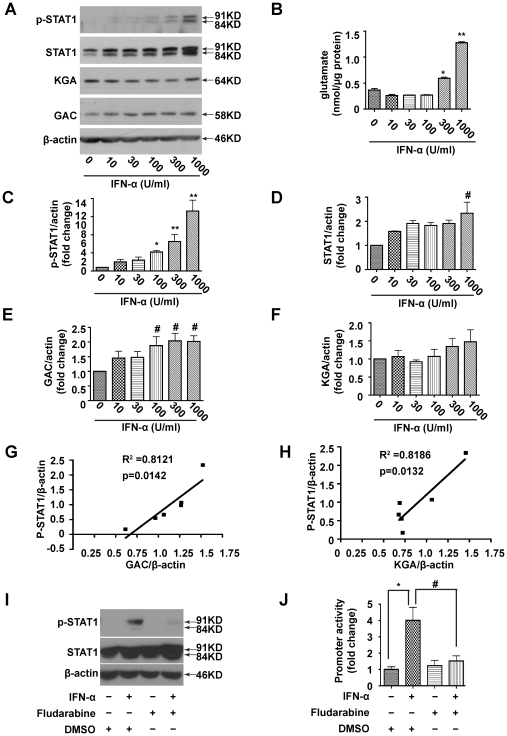
STAT1 phosphorylation is required for IFN-α to activate the GLS1 promoter and induce glutaminase expression and function. (A). MDM were treated with different doses of IFN-α for 24 hours, then p-STAT1 (Tyr 701), STAT1, KGA and GAC were detected by Western blot. β-actin was used as a loading control. (B). Intracellular glutamate was detected using the Amplex^®^ Red Glutamic Acid/Glutamate Oxidase Assay Kit. The data are representative of three independent experiments using three different donors and are the means of triplicate samples. (C-F). Levels of p-STAT1 (C), STAT1 (D), GAC (E) and KGA (F) in Western blot (A) were normalized as a ratio to β-actin and shown as fold change relative to control. Results are shown as the average ± SEM of three independent experiments with three different donors, (G, H). Correlation of p-STAT1 with GAC (G) and p-STAT1 with KGA (H) in representative donor are shown. (I, J). HEK 293T cells were co-transfected with the GLS1 promoter construct and pRL-SV40. 24 hours later, the cells were pretreated with 1 µM fludarabine or 1:10,000 DMSO for 1 hour, then treated with or without 100 U/ml IFN-α for 24 hours. (I). p-STAT1 and STAT1 were detected by Western blot, β-actin was used as a loading control. (J). Luciferase activity in the lysates was measured by luminescence detection. Renilla luciferase was used to normalize transfection efficiency. The data are representative of three independent experiments and are the means of triplicate samples. #, p<0.05. *, p<0.01, **, p<0.001 when compared to control.

IFN-α is known to regulate gene expression through the STAT signaling pathway [24]. To further study the mechanism of IFN-α-activated GLS1 promoter activity, we determined STAT1 phosphorylation following treatment of MDM with varying doses of IFN-α for 24 hours. The STAT1 protein exists as a pair of isoforms, STAT1α (91 kDa) and the splice variant STAT1β (84 kDa). The p-STAT1 and STAT1 antibodies used in our study detected both isoforms. Protein analysis showed that both p-STAT1 (Tyr 701) and total STAT1 were increased by IFN-α in a dose-dependent manner (Fig. 3A, C, D). Similarly, GAC protein levels were upregulated by IFN-α treatment in a dose-dependent manner, whereas KGA was moderately upregulated only at higher doses (300 and 1000 U/ml) of IFN-α treatment (Fig. 3A, E, F). Consistent with the human data, rat astrocytes treated with rat IFN-α had similar upregulation of GAC, KGA (not significant) and total GLS1 mRNA, detected by the specific primers, suggesting that the regulation of glutaminase by IFN-α is not species-specific (Fig. S3). The intracellular glutamate levels, as determined by a glutamate oxidase assay kit, were increased following IFN-α treatment, indicating the upregulated GAC and KGA had functional output to the cells (Fig. 3B). Notably, in MDM, the peak concentration of IFN-α to upregulate GAC and produce glutamate is 1000 U/ml, however, for GLS1 promoter activity detection in 293T cells, the peak concentration is 100 U/ml (Fig. 2B), this inconsistency may be due to their differential expression of type I IFNs receptor IFNR or related signaling intermediates in two different cell models.

To determine the mechanism of glutaminase upregulation, we investigated whether STAT1 phosphorylation is involved in the glutaminase upregulation. STAT1 phosphorylation correlated significantly with the protein levels of GAC and KGA (p<0.01), suggesting the possible involvement of p-STAT1 (Fig. 3G, H). To test whether STAT1 phosphorylation was required in IFN-α-induced glutaminase regulation, we used the chemical inhibitor fludarabine, which specifically inhibits STAT1 phosphorylation [30]. Fludarabine (1μM) pretreatment dramatically decreased (80% reduction) IFN-α-induced STAT1 phosphorylation in HEK 293T cells (Fig. 3I). The weaker p-STAT1β (84 kDa) in 293T cells compared with MDM (Fig. 3A) revealed a cell type-specific expression pattern of the STAT1 isoforms. Following the inhibition of STAT1 phosphorylation, fludarabine significantly decreased the IFN-α-induced GLS1 promoter activity in HEK 293T cells (Fig. 3J); similar data was found in rat GLS1 promoter assays performed in rat astrocytes (Fig. S1D). Collectively, these data demonstrate that IFN-α activates the GLS1 promoter and upregulates glutaminase through STAT1 phosphorylation.

### STAT1 Binds Directly with the GLS1 Promoter in IFN-α Treated Cells

**Figure 4:**
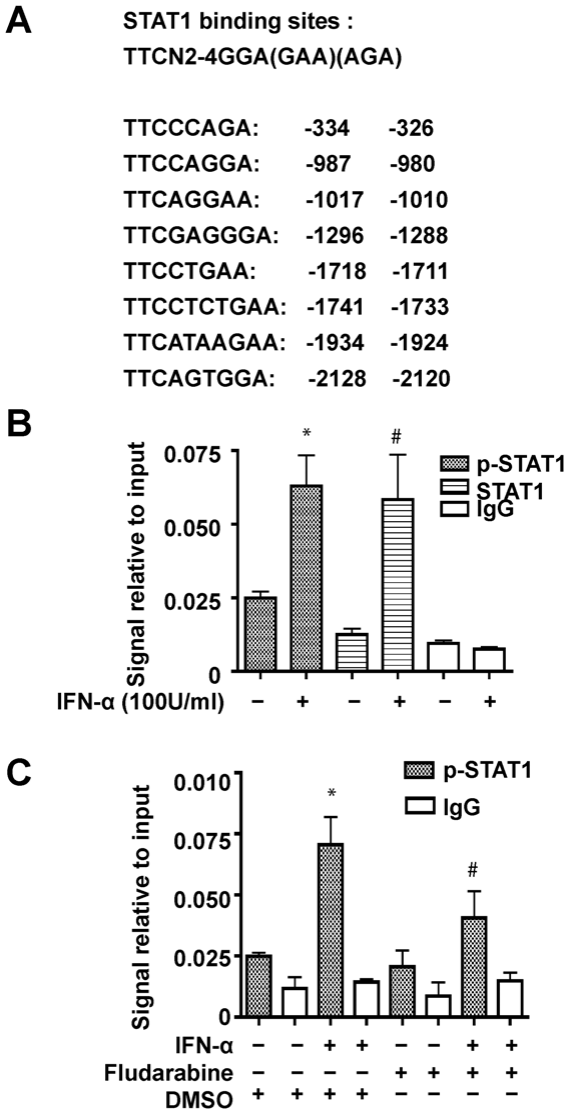
STAT1 binds directly with the GLS1 promoter in IFN-α treated MDM. (A). The predicted STAT1 binding sites in the human GLS1 promoter, TSS is designated as +1. (B). MDM were treated with 100 U/ml IFN-α for 1 hour, then ChIP assay was performed using digested chromatin, p-STAT1 (Tyr 701) and STAT1 antibodies, or IgG antibody as a negative control. Purified DNA was analyzed by quantitative real-time PCR using specific primers. The amount of immunoprecipitated DNA is represented as signal relative to the total amount of input chromatin. The data are representative of three independent experiments using three different donors. #, p<0.05, *, p<0.01 in comparison with control. (C). MDM were pretreated with 1 µM fludarabine or 1:10,000 DMSO for 1 hour, then treated with or without 100 U/ml IFN-α for another hour. ChIP assay was performed using p-STAT1 antibody as in (B). The data are representative of two independent experiments using two different donors. *, p<0.01 in comparison with control that were treated with DMSO only; #, p<0.05 in comparison to cells treated with IFN-α and DMSO.

Since the human GLS1 promoter contains several putative STAT1 binding sites (Fig. 1B, 4A), and STAT1 phosphorylation was found to correlate with IFN-α-induced glutaminase upregulation (Fig. 3), we used a chromatin immunoprecipitation (ChIP) assay to determine the binding of the p-STAT1 to the GLS1 promoter. The rat glutaminase promoter also had several putative STAT binding sites (Fig. S4A). To identify the key promoter sequences that respond to IFN-α, we used serial deletions of the rat promoter construct. To our surprise, even the shortest promoter sequence (−401 +110) responded to IFN-α treatment (Fig. S4B). There were two putative STAT1 binding sites in the shortest promoter construct, which were subsequently targeted for the ChIP assay. We used STAT1 antibody to immunoprecipitate the protein-DNA complex from cellular lysates of rat astroyctes and used RT-PCR to detect the glutaminase promoter sequence. IFN-α dramatically increased GLS1 promoter signaling in the protein-DNA complex, suggesting that there is direct binding of STAT1 with the rat glutaminase promoter (Fig. S4C). Next, in human cells, the putative STAT1 binding site (−334 to −326) that is similarly positioned to one of those we detected in the rat, was used for the ChIP assay. MDM were treated with IFN-α (100 U/ml) for 1 hour and STAT1, p-STAT1 (Tyr701), or IgG antibody was used to immunoprecipitate the protein-DNA complex. Primers specific to the STAT1 binding site located at −334 to −326 were used for real time RT-PCR. The precipitated DNA by STAT1 or p-STAT1 antibody was significantly increased in IFN-α treated cell lysates, but not by the negative control rabbit IgG antibody, indicating that the binding of STAT1 or p-STAT1 with the human GLS1 promoter was significantly increased following IFN-α treatment (Fig. 4B). To determine whether the binding of STAT1 with the human GLS1 promoter was dependent on STAT1 phosphorylation, fludarabine (1 μM) was used to pretreat MDM for 1 hour before the ChIP assay. As expected, fludarabine pretreatment attenuated IFN-α-induced STAT1-GLS1 promoter DNA complex, suggesting that binding of STAT1 to the human GLS1 promoter was dependent on STAT1 phosphorylation (Fig. 4C).

### Increased STAT1 Binding to the GLS1 Promoter in HIV-1 Infected MDM

**Figure 5:**
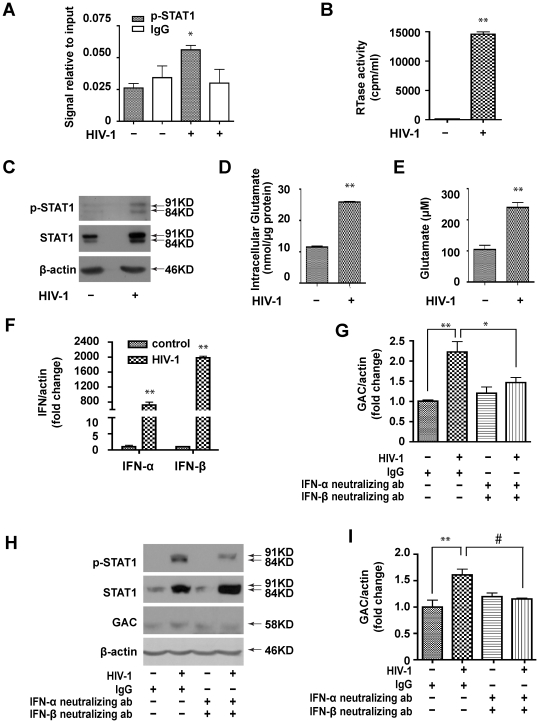
STAT1 binds directly with the GLS1 promoter in HIV-1 infected MDM. (A-E). MDM were infected with or without HIV-1_ADA_ for 5 days. (A). ChIP was performed using p-STAT1 (Tyr 701) antibody; IgG was used as a negative control. (B). Supernatants were tested for RTase activity. (C). p-STAT1 and STAT1 were detected by Western blot with β-actin used as a loading control. (D). Glutamate was detected in supernatants by HPLC. (E). Intracellular glutamate was detected using the Amplex^®^ Red Glutamic Acid/Glutamate Oxidase Assay Kit. The data (A-E) are representative of three independent experiments using three different donors. (F) Human MDM were infected with HIV-1 for 5 days, then total RNA was collected. IFN-α and IFN-β mRNA levels were determined by real-time RT-PCR. Results shown are representative of three independent experiments using three different donors and are means of triplicate samples. (G-I) Human MDM were infected with HIV-1 for 5 days in the presence of IgG or IFN-α/IFN-β neutralizing antibodies. Total RNA and cell lysates were collected and GAC mRNA (G) and protein (H and I) levels were determined by real time RT-PCR and Western blot, respectively. For quantification, GAC expression were normalized as a ratio to β-actin and shown as fold change relative to IgG control . Results (F, I) are shown as the average ± SEM of three independent experiments with three different donors, #, p<0.05, *, p<0.01, **, p<0.001, when compared with uninfected MDM or IgG control.

Our previous study shows that GAC is increased [15] and STAT1 is activated [25] in HIV-infected MDM. To test whether HIV infection induces p-STAT1 binding to the GLS1 promoter and subsequently increases GAC expression, we used a ChIP assay to determine the p-STAT1-GLS1 promoter DNA complex in HIV_ADA_-infected and uninfected MDM. The HIV-1 replication levels were confirmed by RTase assay (Fig. 5B). p-STAT1 was significantly induced to bind to the GLS1 promoter in HIV_ADA_-infected MDM (Fig. 5A). This increase of the p-STAT1-GLS1 promoter DNA complex upon HIV-1 infection was associated with a four-fold and two-fold increase of p-STAT1 and STAT1 protein levels, respectively, compared with uninfected cells (Fig. 5C). Consequently, both intracellular (Fig. 5D) and extracellular glutamate levels (Fig. 5E) were increased, indicating that increased p-STAT1 binding to the GLS1 promoter might contribute to the excess levels of glutamate seen in HIV-1-infected MDM. To further determine the mechanism of STAT1 activation in HIV-1-infected MDM, we detected the expression of type I IFNs and found that both IFN-α and IFN-β mRNA levels were significantly increased in HIV-1 infected MDM (Fig. 5F). More importantly, IFN-α and IFN-β neutralizing antibodies, which were able to block the binding of type I IFNs with their receptor IFNR and the downstream STAT1 phosphorylation (60% reduction, [25] Fig. 5H), significantly attenuated both mRNA (Fig. 5G) and protein levels (Fig. 5H and I) of GAC induced by HIV-1 infection. Together, these results demonstrate that HIV-1 induces type I IFNs response, which in turn activates STAT1 and induces gene transcription of glutaminase.

### Significant Correlation Between STAT1, IFN-α, IFN-β and GAC mRNA Levels in a Set of Brain Tissues Including HAD Patients

**Figure 6:**
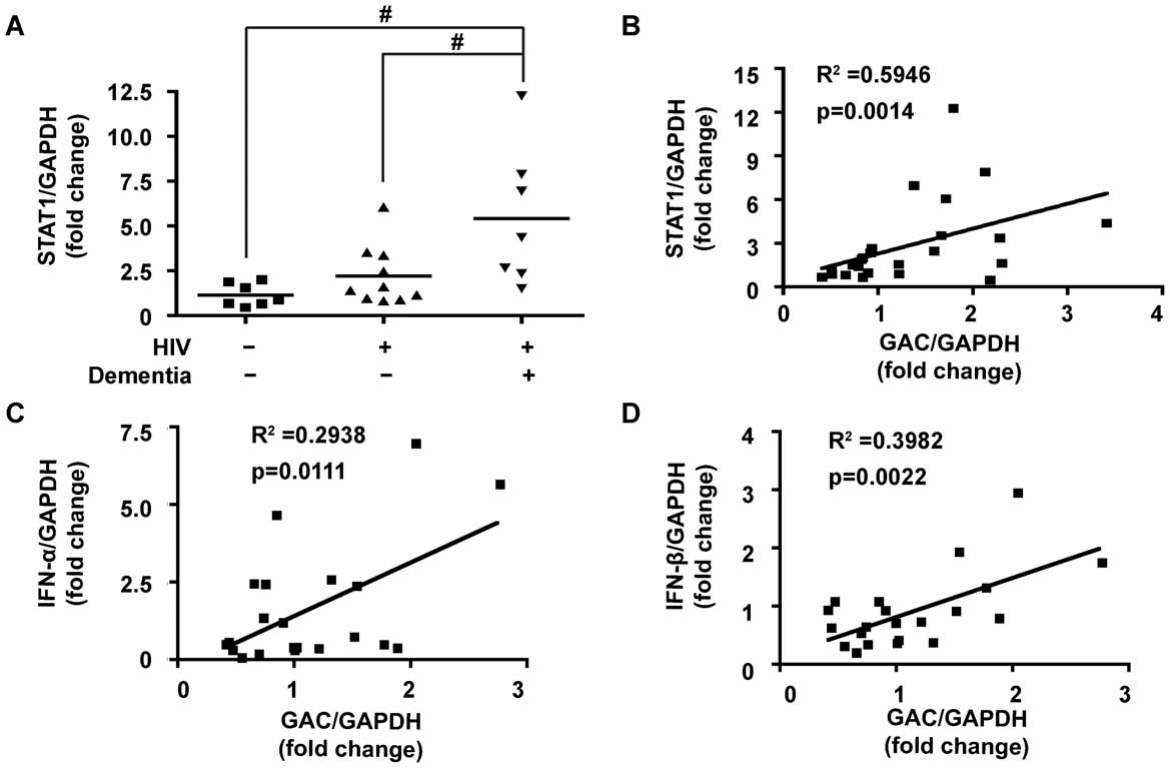
Significant correlation between STAT1 and GAC mRNA levels in brain tissues of HAD patients. Total RNA from post-mortem brain tissue collected from HAD patients, HIV serum-positive patients without dementia, and HIV serum negative individuals were extracted and subjected to real time RT-PCR for STAT1, GAC, IFN-α2 and IFN-β. GAPDH expression was used as an internal control. (A) Levels of STAT1 are normalized as a ratio to GAPDH and shown as fold change relative to the average of HIV serum negative controls. Results are shown as the average ± SEM, #, p<0.05. Correlation of GAC with STAT1 (B), IFN-α2 (C) and IFN-β (D) mRNA levels was determined by Spearman correlation.

*T*o further investigate the clinical relevance of STAT1 and GLS1 to HAD, we analyzed STAT1 and GLS1 mRNA levels from post-mortem brain tissue collected from HAD patients, HIV serum-positive patients without dementia, and HIV serum negative individuals. We have previously reported that protein levels of GAC and STAT1 were significantly increased in HAD brain tissues compared with HIV serum negative individuals [9]. Using the mRNA from the same sets of brain tissues, we found that STAT1 mRNA levels were significantly increased in HAD patients compared to HIV serum negative individuals and HIV serum-positive patients without dementia (p<0.05) (Fig. 6A). Furthermore, significant correlations were found between GAC and STAT1 (p = 0.0014), IFN-α (p = 0.0111), and IFN-β (p = 0.0022) (Fig. 6B, C and D). This clinical relevance of STAT1 and type I IFNs in association with GAC strongly indicates that STAT1 and type I IFNs play important roles in GAC expression in HAD.

## Discussion

Little is known about how glutaminase expression is regulated. In our study, we used a dual-luciferase reporter assay system to demonstrate that IFN-α specifically activated the GLS1 promoter (Fig. 2, S1). The elevated GLS1 promoter activity correlated with increased levels of GLS1 mRNA and protein (Fig. 3, S3). Furthermore, increased glutaminase expression by IFN-α treatment was correlated with STAT1 phosphorylation and could be reduced by fludarabine, a chemical that inhibits STAT1 phosphorylation (Fig. 3). In a well-characterized model of HIV-1 infection in human MDM, we have shown that IFN-α treatment or HIV-1 infection of MDM activates STAT1 phosphorylation to bind to and upregulate the GLS1 promoter activity, and subsequently increased glutaminase and glutamate production (Fig. 4, 5). Importantly, blocking type I IFNs with neutralizing antibodies in HIV-1-infected MDM blocked GAC upregulation at both mRNA and protein levels (Fig. 5G, H and I), confirming the critical role of type I IFNs on HIV-1-induced glutaminase upregulation. These data suggest that IFN-α phosphorylates and activates STAT1; STAT1 binds with GLS1 promoter and enhances promoter activity, which increases glutaminase gene transcription. Since glutaminase converts glutamine into glutamate, which is a neurotoxin when in excess levels, IFN-α may have adverse effects through glutamate-mediated excitotoxicity in addition to its traditional cytotoxic activity in antigen presenting cells. Therefore, we propose that the findings here may have potential implications for understanding the pathogenesis of HAND, particularly the neurotoxic processes that involve significant production of inflammatory cytokines and soluble factors including glutamate.

The clinical relevance of this observation is further corroborated with the investigation of post-mortem brain tissues. The tissue GAC mRNA levels were significantly correlated with STAT1, IFN-α and IFN-β (Fig. 6). These important data extend our previous observation that GAC is correlated with STAT1 [9] and the physiological indication of this finding is significant because elevated STAT1 and type I IFNs levels are not only observed in HAD, but also in many other neurodegenerative diseases [31], [32], [33], [34] though the exact functions of STAT1 and type I IFNs in diseases remains to be elucidated. In addition to STAT1 and type I IFNs, dysregulated glutaminase has also been indicated in a few animal models of neurodegenerative diseases. Microglia glutaminase has been suggested to have neurotoxic potentials in MECP2 knockout mice, which underlined the potential pathogenic role of dysregulated glutaminase in the CNS [35]. Because IFNs are released in the late stage of HIV-1 infection [36], [37], and HIV-1 infection could also increase glutaminase expression and glutamate production [31], [32], the identification of type I IFNs and STAT1 as key regulators of glutaminase expression could provide critical links between HIV-1 infection, innate immune response and the dysregulation of glutaminase in infected macrophages and microglia.

Eukaryotic transcription and translation regulation are complex. For glutaminase, the control of expression may occur at the transcriptional, post-transcriptional, translational or post-translational level. In an attempt to characterize the GLS1 promoter regulatory sequences, we cloned the promoter region, up to 2490 bp upstream of the transcription initiation site (TSS) (Fig. 1). Surprisingly, this extensive region contains many putative binding sites for STAT1, indicating potential STAT1-mediated regulation of the glutaminase promoter activity. As expected, IFN-α, which is known to increase STAT1 phosphorylation and nuclear translocation, specifically upregulated GLS1 promoter activity (Fig. 2, S1). Previously, it was reported that longer 16×GCA microsatellite alleles in the promoter region correlate with higher activity of glutaminase *in vivo* [38]. In addition, acidic medium increases glutaminase expression through stabilization of KGA mRNA [28]. The 3′-UTR of KGA mRNA contains a direct repeat of an 8 nucleotide, AU-rich sequence that functions as a pH-response element (pHRE), which binds multiple RNA-binding proteins, to impart a pH-responsive stabilization and to thereby protect the KGA mRNA [39], [40]. Moreover, c-Myc suppression of miR-23 has been identified as a mechanism to enhance glutaminase expression through post-transcriptional regulation [41]. To our knowledge, this is the first report demonstrating that glutaminase transcription and function are under the control of STAT1 and type I IFNs.

The presence of multiple transcripts for glutaminase genes and the simultaneous expression of several different glutaminase isoforms reported in mammalian tissues and cells indicate that post-transcriptional splicing also exists [13], [14], [42]. However, exactly how splicing occurs and the overall significance of the glutaminase isoforms is still unclear. GAC regulation has recently been observed in a variety of tumors [43], indicating that the GAC isoform is possibly an important component of tumor cells, and modulation of GAC activity may provide a therapeutic avenue for cancer.

Cytokines typically regulate gene promoters through directing transcription factors to their binding sites in the promoter region. The proinflammatory cytokine IL-6 upregulates calcium-sensing receptor gene transcription via STAT1/3 [44]. IFN-γ upregulates MUC4 in pancreatic cancer cells through STAT1 expression [45]. Notably, cytokines other than type I IFNs have been reported to regulate glutamate metabolism. TNF-α induces neurotoxicity by inhibiting glutamate uptake through the glutamate transporter of astroglial cells [46] as well as by upregulating glutaminase with concurrent release of glutamate through hemichannels in activated mouse microglia [47]. On the other hand, TGF-β increases expression of glutaminase in a porcine kidney cell line [28]. In our study we found that IFN-α specifically activated the GLS1 promoter through the STAT1 pathway in human macrophages [48]. Regulation of the GLS1 promoter by IFN-α or HIV-1 infection is specific to STAT1 because when we used STAT3 antibody in the ChIP assay we found that neither IFN-α nor HIV-1 infection significantly increased binding between STAT3 and the GLS1 promoter (data not shown). Furthermore, STAT1 plays an important role in the cellular response to IFN-α [49]. In our study STAT1 binds to the GLS1 promoter to increase glutaminase for glutamate production in HIV-1 infected MDM (Fig. 5D, E). Although there are multiple predicted STAT1 binding sites in both the rat and human GLS1 promoter (Fig. 4A and S2A), in this study we focused on the two sites that are closest to the translation start site (Fig. 4B, C, Fig. 5A and Fig. S3C). The function of other STAT1 putative binding sites needs to be further investigated.

The doses of IFN-α used in our *in vitro* experiments were between 10 and 1000 U/ml. It is possible that these concentrations are higher than that would be detected in blood. However, high plasma levels of IFN-α can be detected during acute HIV infection [48]. Furthermore, it is likely that much HIV-1 pathogenesis occurs in the tissue where type I IFNs may be more concentrated than in the circulatory system. Our data demonstrate that IFN-α, at concentrations as low as 10 U/ml, was sufficient to increase phosphorylated STAT1 (Fig. 3A and C), promoter activity (Fig. 2B) and GAC expression (Fig. 3A and C) compared with control. Therefore, we used IFN-α at concentrations between 10 and 1000 U/ml for a dose-response curve and chose 100 U/ml as a representative dose for treatment throughout the text.

In conclusion, we found that IFN-α activates STAT1 to bind to and activate the GLS1 promoter in HIV-1-infected macrophages. This binding results in the upregulation of glutaminase and a subsequent increase in glutamate production, which may drive the excitotoxicity of neurons during HIV infection. We propose this finding has potential implications for understanding the pathogenesis of HAND. Dysregulated glutaminase and its potential neurotoxic process stemming from innate immune activation raise important questions about how to regulate the innate immune response in an effective and specific way without harming the vulnerable neuronal tissues with excitotoxicity.

## Materials and Methods

### Reagents

Recombinant proteins and chemicals were obtained as follows: IFN-α2, IFN-β and IFN-α neutralizing antibody (PBL Interferon Source, Piscataway, NJ); IL-1β, IL-6, IL-10, TNF-α, IFN-γ, Trail, and IFN-β neutralizing antibody (R&D systems. Minneapolis, MN, USA); and fludarabine (Sigma-Aldrich, St, Louis, MO, USA).

### Plasmid Construct

Two overlapping segments with varying levels of GC content of the human GLS1 promoter were amplified by PCR directly from human bacterial artificial clone (BAC) PR11-413B20 (Invitrogen, Carlsbad, CA) using *PfuUltra*™ II Fusion HS DNA Polymerase (Agilent Technologies, Santa Clara, CA) with the primers: 5′-CGGGGTACCGGAGCAAAAAGGAAGTCGAAGAGTAGATCTGACAACCCAACCATAG-3′ (sense for low GC content section), 5′-AAATGTACACCGATACTTCAAAAATAGTTTTGAATAGCAATGATTCCGTTAACGC-3′ (antisense for low GC content section), 5′-CGGTGTACATTTAAAAAAAATAATTTTTAAAAAAGAAAGCTGTCGTATTCTGGATTCCGC-3′ (sense for GC rich section), 5′-CCGCTCGAGGCCGCCGGGTCCGTCAGCGCCCGCTCAACAGGGGAGGATGCTCC-3′ (antisense for GC rich section). The BAC plasmid was initially denatured at 95°C for 3 min, followed by 30 cycles of denaturing at 94°C for 30 s, primer annealing at 61°C for 30 s, and elongation at 72°C for 4 min. Taq DNA polymerase (Roche, Indianapolis, IN, USA) was used to add poly A for ligating the PCR products to the pGEM-T easy vector with T4 ligase (Promega, Madison, WI, USA). After the sequence was confirmed, the two segments with varying levels of GC content were cut and ligated to the pGL-3 vector (Promega) using the specific restriction enzymes, KpnI, BsrGI and XhoI (New England Biolabs, Ipswich, MA, USA).

### Cell Culture, Transfection and Luciferase Reporter Assay

HEK 293T (ATCC, Mansassas, VA, USA) cells were cultured in 24-well plates in Dulbecco’s modified Eagles medium (DMEM, GIBCO Invitrogen Corp, Carlsbad, CA) with 10% heat-inactivated fetal bovine serum (FBS) (GIBCO Invitrogen Corp) and an antibiotic mixture containing penicillin and streptomycin. 24 hours after plating, cells were transfected with the pGL3-basic or GLS1 promoter-driven Firefly luciferase reporter plasmid with Lipofectamine^TM^ LTX and PLUS reagent (Invitrogen, Carlsbad, CA). Cells were co-transfected with 5 ng of Simian Virus 40 promoter-driven Renilla luciferase (pRL-SV40) plasmid as a control for transfection efficiency. 24 hours post-transfection, cells were treated with cytokines for another 24 hours; then the Firefly and Renilla luciferase were analyzed using a Dual-Luciferase Reporter System (Promega) according to the manufacturer’s instructions.

### MDM and HIV-1 Infection

Human monocytes were cultured as adherent monolayers at a density of 1.1 × 10^6^ cells/well in 24-well plates and cultivated in Dulbecco’s modified Eagles medium (DMEM, GIBCO Invitrogen Corp) with 10% heat-inactivated pooled human serum (Cambrex Bio Science, Walkersville, MD, USA), 50 µg/ml gentamicin, 10 µg/ml ciprofloxacin (Sigma-Aldrich) and 1000 U/ml highly purified recombinant human macrophage colony stimulating factor (MCSF) (a generous gift from Wyeth Institute, Cambridge, MA, USA). Seven days after plating, MDM were infected with laboratory HIV-1_ADA_ strain at a multiplicity of infection (MOI) of 0.1 virus/target cell. The HIV-1_ADA_ was isolated from the peripheral blood mononuclear cells (PBMCs) of an infected patient with Kaposi’s sarcoma [50]. For virus stock preparation, supernatants of HIV-1_ADA_-infected MDM were collected. The titers of the virus in the supernatants were determined as we previously described [51]. For HIV-1 infection, viral stocks were diluted into the desired MOI for overnight incubation with MDM. On the second day, medium was removed and substituted with MDM culture medium that was half-exchanged every two days. Stock virus was screened for mycoplasma and endotoxin using hybridization and *Limulus* amebocyte lysate assays, respectively. Five days after infection, HIV-1-infected and replicated uninfected MDM were harvested for ChIP and Western blot assays. Culture supernatants were obtained and subsequently stored at -80°C until a reverse transcriptase (RTase) assay could be performed.

### Western Blot Analysis

Cell lysates from MDM or HEK 293T cells were prepared with M-PER Mammalian Protein Extraction Buffer (Pierce, Rockford, IL, USA). Protein concentration was determined using the BCA Protein Assay Kit (Pierce). Protein (20 µg) was electrophoresed on pre-cast 10% SDS-PAGE gels (Bio-Rad, Hercules, CA) and transferred to an Immuno-Blot PVDF membrane (Bio-Rad). Proteins on the membrane were incubated with purified primary antibodies for total STAT1 or phospho-STAT1 (p-STAT1, Tyr701) (Cell Signaling Technologies, Danvers, MA, USA), for GAC or KGA (Dr. N. Curthoys, Colorado State University), or for β-actin (Sigma-Aldrich) overnight at 4^o^C followed by a horseradish peroxidase-ligand secondary anti-rabbit or anti-mouse (1:10000 dilution; Cell Signaling Technologies) antibody for 1 h at room temperature. Antigen-antibody complexes were visualized by enhanced chemiluminescence (Amersham Biosciences, Piscataway, NJ, USA) and captured with CL-X Posure™ Film (Pierce). For data quantification the films were scanned with a CanonScan 9950F scanner; the acquired images were then analyzed on a Macintosh computer using the public domain NIH image program (developed at the U.S. National Institutes of Health and available on the internet at http://rsb.info.nih.gov/nih-image/).

### ChIP Assay

ChIP assay was performed using a SimpleChIP® Enzymatic Chromatin IP Kit (#9003, Cell Signaling Technologies) according to the manufacturer’s instructions. Quantitative PCR was performed using one pair of primers corresponding to the STAT1 binding site located at -598 in the human GLS1 promoter: 5'-CCTGCCAAACCTGCTGACC-3' (sense), 5'-CCACTCTGGGAATTCGAG-3' (antisense). Quantifications were normalized to input.

### Measurements of HIV-1 RTase

RT activity was determined in triplicate samples of cell culture fluids. For this assay, 10 µl of supernatant was incubated in a reaction mixture of 0.05% Nonidet P-40, 10 µg of poly(A)/ml, 0.25 µg of oligo(dT)/ml, 5 mM dithiothreitol, 150 mM KCl, 15 mM MgCl_2_, and [^3^H]TTP in Tris-HCl buffer (PH 7.9) for 24 hours at 37°C. Radiolabeled nucleotides were precipitated with cold 10% trichloroacetic acid on paper filters in an automatic cell harvester and washed with 95% ethanol. Radioactivity was estimated by liquid scintillation spectroscopy [52].

### Intracellular and Extracellular Glutamate Analysis

Intracellular glutamate detection was performed with the Amplex^®^ Red Glutamic Acid/Glutamate Oxidase Assay Kit from Invitrogen following the manufacture’s procedure. HPLC analysis for extracellular glutamate was performed as previously described [8].

### RT-PCR for Detection of Human GLS1, IFN-α and IFN-β

Total RNA was isolated with TRIzol Reagent (Invitrogen Corp) and RNeasy Kit according to the manufacturer's protocol (Qiagen Inc.). Primers used for real-time RT-PCR include human STAT1 (Hs01014002), human GAC (ID #528445: the forward sequence was 5′-TATGGAAAAAAGTGTCACCTGAGTCA-3′, the reverse sequence was 5′-GCTTTTCTCTCCCAGACTTTCCATT-3′, and the probe sequence was 5′-AATGAGGACATCTCTACAACTGTA-3′), human IFN-α2 (Hs00265051_S1), human IFN-β1 (Hs01077958S1_S1), human GAPDH (part #4310884E), human β-actin (part #4333762), rat GLS1 (00561285_m1), rat KGA (Rn01640312_m1), rat GAC (ID #792159: the forward sequence was 5′-CTTTGGACCATTGGACTATGAGAGT-3′, the reverse sequence was 5′-AGGTGACACTTTTTTCCACACTGT-3′, and the probe sequence was 5′-CTCCAGCAAGAACTTG-3′) and rat GAPDH (Rn99999916_s1) from Applied Biosystems Inc. Real-time RT-PCR was performed using the one-step quantitative TaqMan assay in a StepOne Real-Time PCR system (Applied Biosystems Inc.). Relative STAT1, GAC, IFN-α2, and IFN-β1 mRNA levels were determined and standardized with a GAPDH or β-actin internal control using comparative ΔΔCT method. All primers used in the study were tested for amplification efficiencies and the results were similar.

### Statistical Tests

Data was analyzed as means ± standard deviation unless otherwise specified. The data were evaluated statistically by the analysis of variance (ANOVA), followed by the Tukey-test for paired observations. Significance was considered to be less than 0.05 unless otherwise specified. All assays were performed at least three times with triplicate or quadruplicate determinations for each.

### Ethics Statement

Primary rat astrocytes were made from embryonic day 14–15 rat embryos in accordance with ethical guidelines for care and use of laboratory animals set forth by the National Institutes of Health (NIH), with Institutional Animal Care and Use Committee (IACUC) #: 04-097-01; MDM were used in full compliance with the University of Nebraska Medical Center and NIH ethical guidelines, with the Institutional Review Board (IRB) #: 162-93-FB. We have the informed written consent from all participants involved in this study.

## Supporting Information

Figure S1 **IFN-α specifically activates rat GLS1 promoter.** Rat astrocytes were co-transfected with the rat GLS1 promoter construct and pRL-SV40. 24 hours later, the cells were treated with either various individual cytokines for another 24 hours (A), IFN-α of varying doses for 24 hours (B), or with 100 U/ml IFN-α for varied time lengths (C). In (A), 100 U/ml IFN-α, 100 U/ml IFN-β, 100 ng/ml IFN-γ, 10 ng/ml IL-1β, 10 ng/ml IL-6, 50 ng/ml TRAIL, or 50 ng/ml TNF-α was used. (D). 24 hours after transfection, cells were pretreated with 1 µM fludarabine or 1:10,000 DMSO for 1 hour then treated with IFN-α (100 U/ml) for 24 hours. Luciferase activity in the lysates was measured by luminescence detection. Renilla luciferase was used as internal control to normalize transfection efficiency. The data are representative of three independent experiments and are the means of triplicate samples. #, p<0.05, *, p<0.01, **, p<0.001 in comparison to control.
(TIF)

Figure S2 **Type I IFN neutralizing antibodies block IFN-α-induced GLS1 promoter activation.** HEK 293T cells were co-transfected with the human GLS1 promoter construct and pRL-SV40. 24 hours later, the cells were pre-treated with IgG or IFN-α/IFN-β neutralizing antibodies for 1 hour then treated with IFN-α (100 U/ml) for 24 hours. Luciferase activity in the lysates was measured by the luminescence detection. Renilla luciferase was used as internal control to normalize transfection efficiency. The data are representative of two independent experiments and are the means of triplicate samples. #, p<0.05.
(TIF)

Figure S3 **glutaminase mRNA was increased in IFN-α treated rat astrocytes.** Rat astrocytes were treated with the indicated doses of IFN-α for 24 hours. Real-time RT-PCR was used to detect KGA, GAC or total GLS1. KGA, GAC or total GLS1 expression were normalized to GAPDH and shown as fold change relative to the untreated control. Results are shown as the average ± SEM of two independent experiments with two different donors. #, p<0.05, *, p<0.01, **, p<0.001 when compared to control.
(TIF)

Figure S4 **STAT1 binds directly with the GLS1 promoter in IFN-α treated rat astrocytes.** (A). The predicted STAT1 binding sites in the rat GLS1 promoter. TSS is designated as +1. (B) Rat astrocytes were co-transfected with rat GLS1 promoter constructs with serial deletion and pRL-SV40. 24 hours later, the cells were treated with 100 U/ml IFN-α for 24 hours. Luciferase activity was measured and analyzed. The data are representative of three independent experiments with three different donors. *, p<0.01, **, p<0.001 in comparison to control. (C). Rat astrocytes were treated with 100 U/ml IFN-α for 1 hour, then ChIP assay was performed using STAT1 antibody. IgG antibody was used as a negative control, whereas histone H3 antibody was used as a positive control. Purified DNA was amplified by PCR using specific primers. The PCR products were analyzed on 2% agarose gel.
(TIF)
