# Supplementary figures and images for: Interferon-α Regulates Glutaminase 1 Promoter through STAT1 Phosphorylation: Relevance to HIV-1 Associated Neurocognitive Disorders
Source: PLoS One. 2012 Mar 30;7(3):e32995. doi: 10.1371/journal.pone.0032995 (PMC3316554; doi:10.1371/journal.pone.0032995)

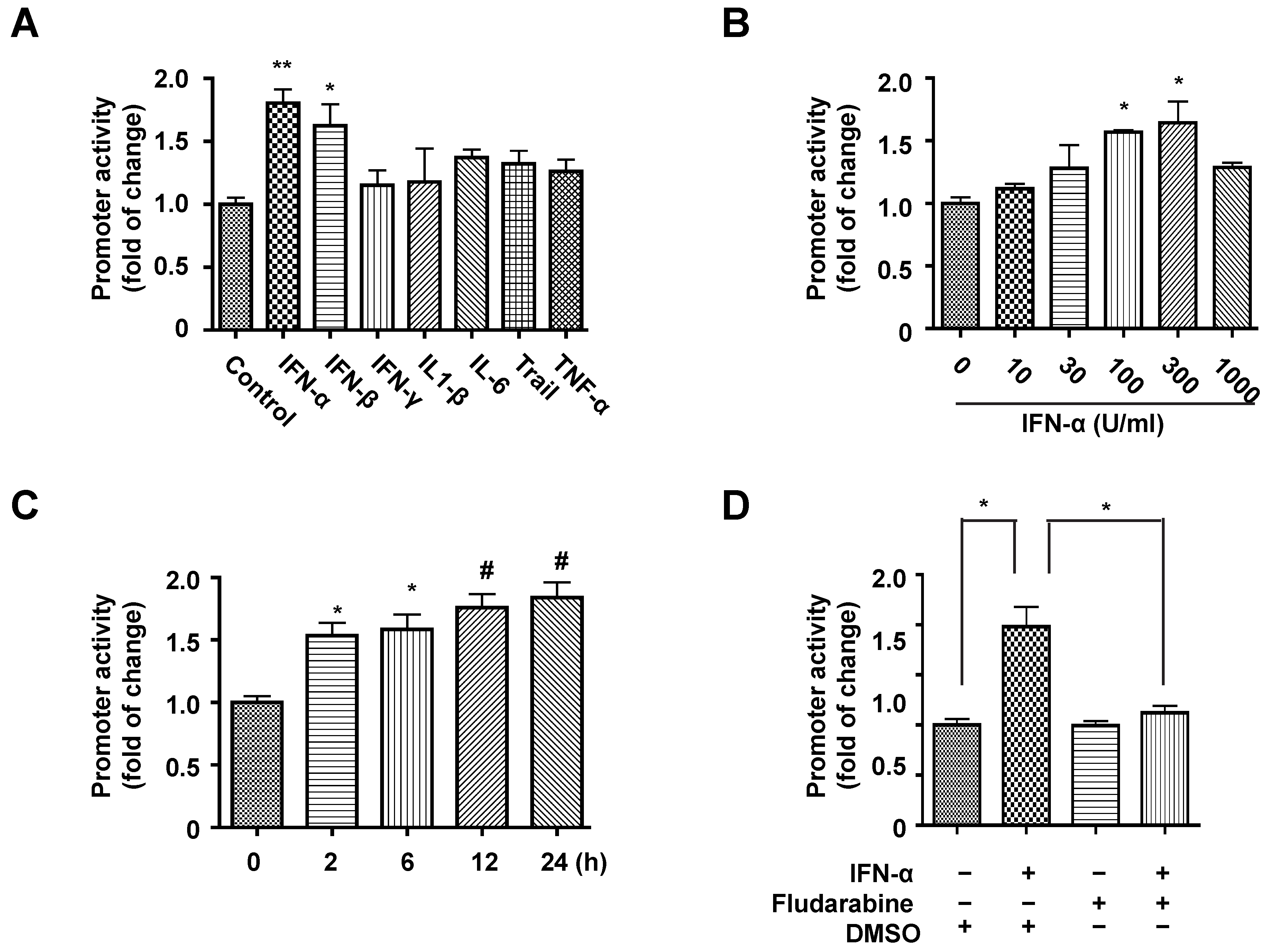

Supplement: Figure S1 — IFN-α specifically activates rat GLS1 promoter. Rat astrocytes were co-transfected with the rat GLS1 promoter construct and pRL-SV40. 24 hours later, the cells were treated with either various individual cytokines for another 24 hours (A), IFN-α of varying doses for 24 hours (B), or with 100 U/ml IFN-α for varied time lengths (C). In (A), 100 U/ml IFN-α, 100 U/ml IFN-β, 100 ng/ml IFN-γ, 10 ng/ml IL-1β, 10 ng/ml IL-6, 50 ng/ml TRAIL, or 50 ng/ml TNF-α was used. (D). 24 hours after transfection, cells were pretreated with 1 µM fludarabine or 1:10,000 DMSO for 1 hour then treated with IFN-α (100 U/ml) for 24 hours. Luciferase activity in the lysates was measured by luminescence detection. Renilla luciferase was used as internal control to normalize transfection efficiency. The data are representative of three independent experiments and are the means of triplicate samples. #, p<0.05, *, p<0.01, **, p<0.001 in comparison to control. (TIF) [file pone.0032995.s001.tif]

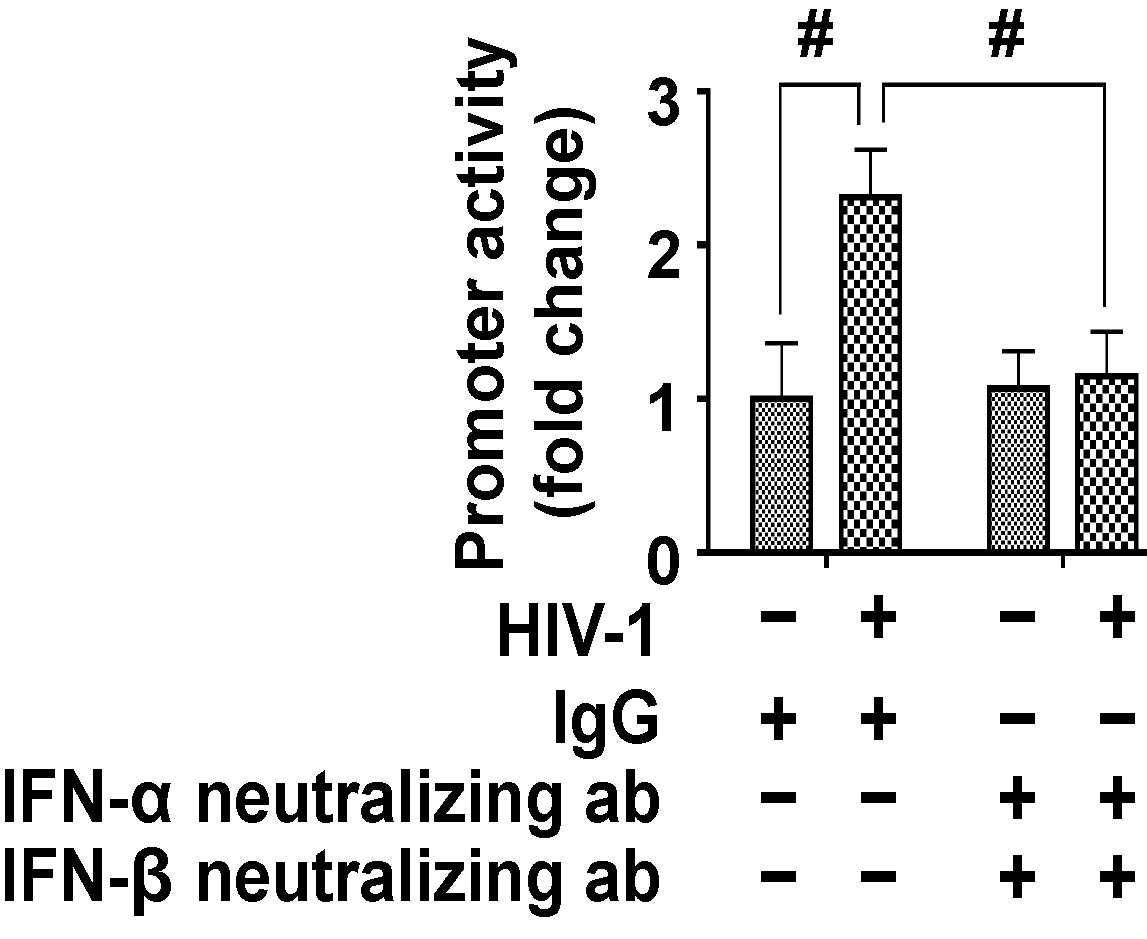

Supplement: Figure S2 — Type I IFN neutralizing antibodies block IFN-α-induced GLS1 promoter activation. HEK 293T cells were co-transfected with the human GLS1 promoter construct and pRL-SV40. 24 hours later, the cells were pre-treated with IgG or IFN-α/IFN-β neutralizing antibodies for 1 hour then treated with IFN-α (100 U/ml) for 24 hours. Luciferase activity in the lysates was measured by the luminescence detection. Renilla luciferase was used as internal control to normalize transfection efficiency. The data are representative of two independent experiments and are the means of triplicate samples. #, p<0.05. (TIF) [file pone.0032995.s002.tif]

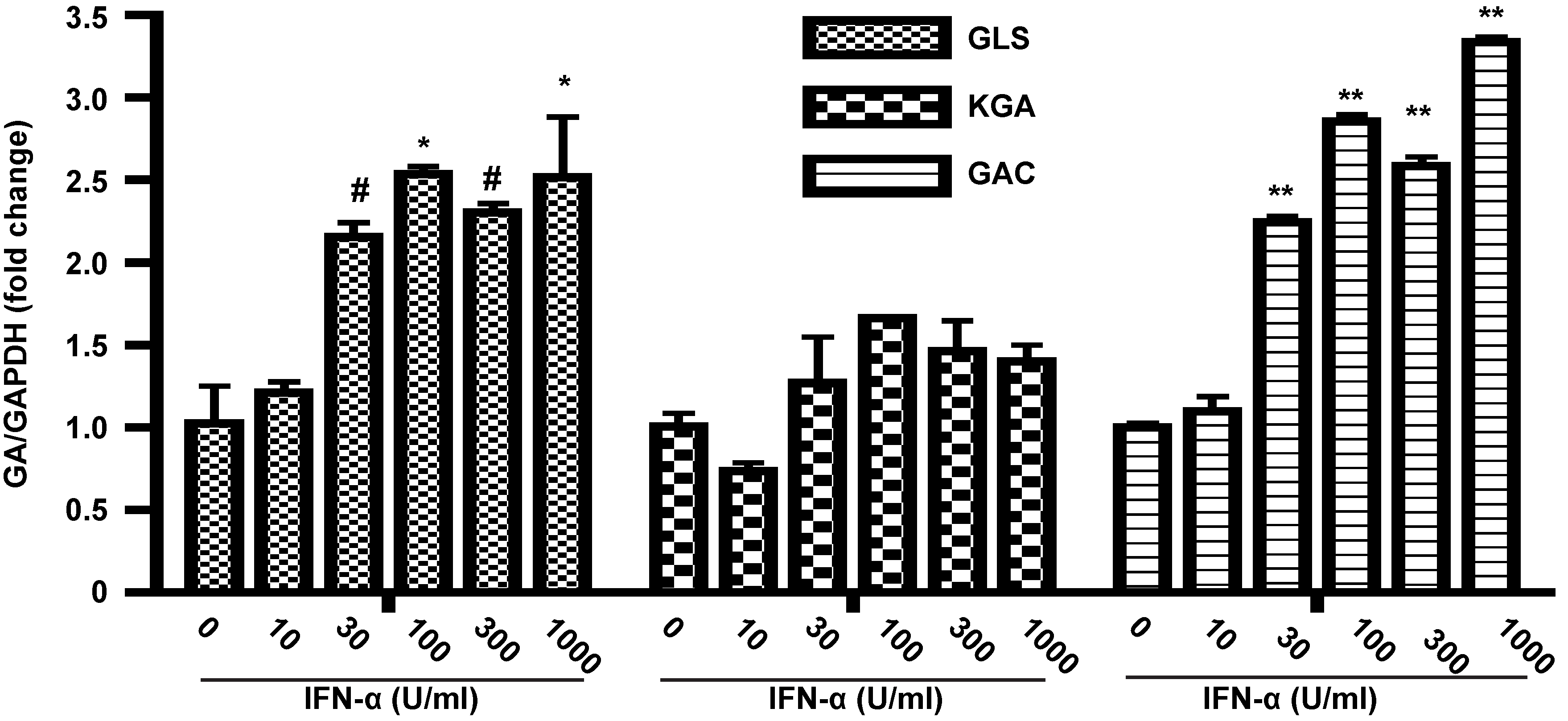

Supplement: Figure S3 — glutaminase mRNA was increased in IFN-α treated rat astrocytes. Rat astrocytes were treated with the indicated doses of IFN-α for 24 hours. Real-time RT-PCR was used to detect KGA, GAC or total GLS1. KGA, GAC or total GLS1 expression were normalized to GAPDH and shown as fold change relative to the untreated control. Results are shown as the average ± SEM of two independent experiments with two different donors. #, p<0.05, *, p<0.01, **, p<0.001 when compared to control. (TIF) [file pone.0032995.s003.tif]

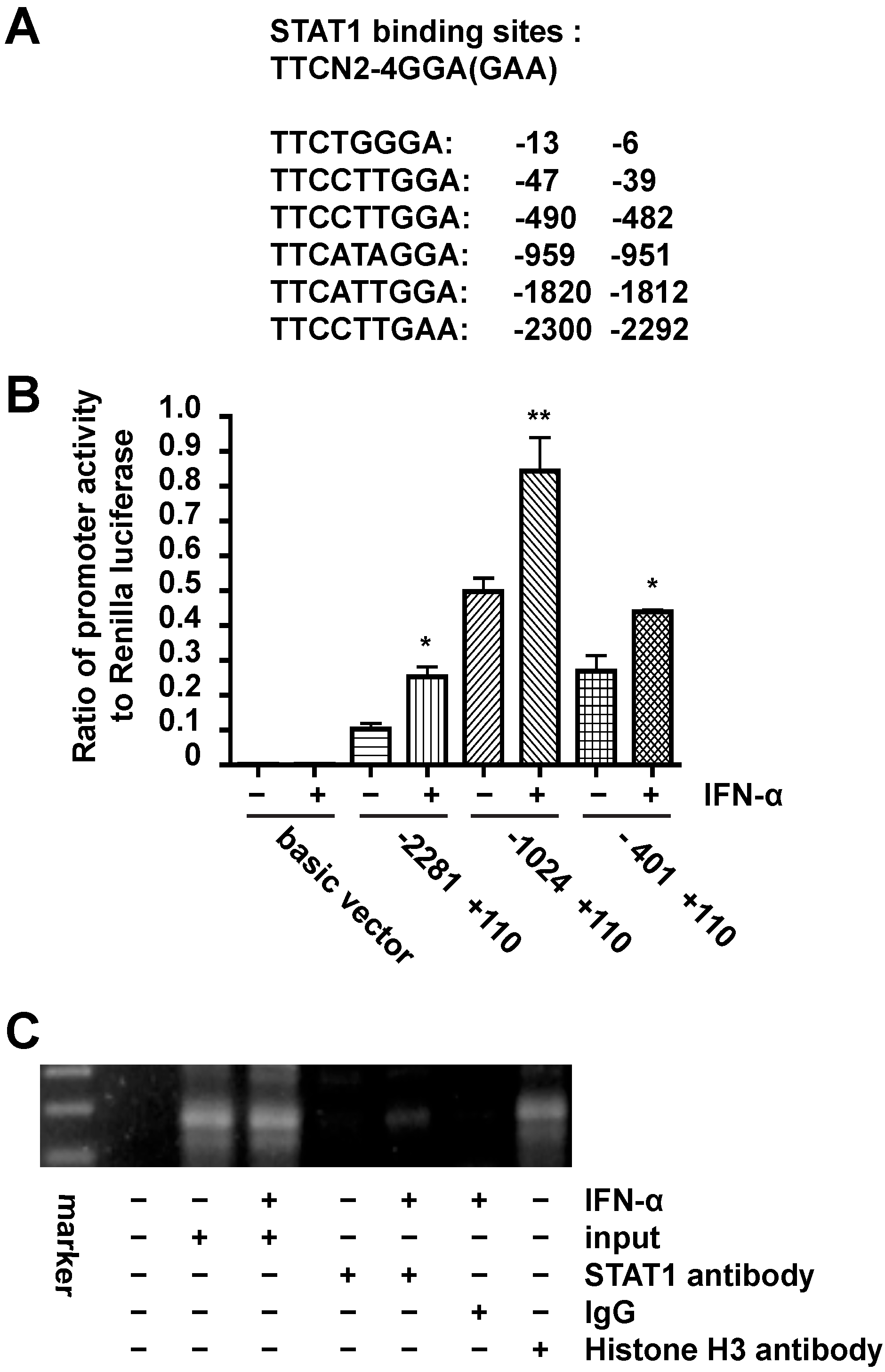

Supplement: Figure S4 — STAT1 binds directly with the GLS1 promoter in IFN-α treated rat astrocytes. (A). The predicted STAT1 binding sites in the rat GLS1 promoter. TSS is designated as +1. (B) Rat astrocytes were co-transfected with rat GLS1 promoter constructs with serial deletion and pRL-SV40. 24 hours later, the cells were treated with 100 U/ml IFN-α for 24 hours. Luciferase activity was measured and analyzed. The data are representative of three independent experiments with three different donors. *, p<0.01, **, p<0.001 in comparison to control. (C). Rat astrocytes were treated with 100 U/ml IFN-α for 1 hour, then ChIP assay was performed using STAT1 antibody. IgG antibody was used as a negative control, whereas histone H3 antibody was used as a positive control. Purified DNA was amplified by PCR using specific primers. The PCR products were analyzed on 2% agarose gel. (TIF) [file pone.0032995.s004.tif]
